# 肺癌外科手术进展

**DOI:** 10.3779/j.issn.1009-3419.2013.03.11

**Published:** 2013-03-20

**Authors:** Mark W. Hennon, Sai Yendamuri, 燕 丁, 娟 南

**Affiliations:** 1 Department of Toracic Surgery, Roswell Park Cancer Institute, Surgery, State University of New York, Buffalo, NY, USA; 2 天津医科大学总医院，天津市肺癌研究所，天津市肺癌转移与肿瘤微环境重点实验室

**Keywords:** 肺癌, 预后, 胸腔镜

## Abstract

近年来微创技术在各期肺癌的检测、诊断和治疗中的应用出现爆发。这些技术的应用提高了手术的风险-收益比，并且使考虑肺癌手术治疗的患者更易接受手术。同时它们为晚期肺癌患者综合治疗的实施提供了便利。该综述总结了代表肺癌胸外科手术前沿的现有外科技术。

## 前言

对于准备接受手术治疗的非小细胞肺癌(nonsmall cell lung cancer, NSCLC)患者来说，现在是一个令人激动的时刻。用于NSCLC的诊断、分期、切除以及姑息治疗的新方法和技术不断涌现和完善。针对NSCLC患者的治疗策略日益复杂，在精确的临床和外科分期的驱动下，这些新的技术和工具可实现治疗计划的最优化。适用于未确诊肺结节的外科入路的工具也在发展。肺外科微创技术的进步降低了与解剖性肺切除相关的发病率，并扩展了胸腔镜切除的适应症。随着这些技术的改进，更高分期的肺癌患者可安全接受胸腔镜切除，术后发病率降低，这或可提高他们完成辅助治疗的机会。最后，不能接受解剖性肺叶切除术或肺切除术的不可切除的患者有了更多可有效治疗疾病的姑息治疗选择。本综述将总结代表肺癌胸外科手术前沿的研究。

## 肺癌诊断技术进展

诊断工具的重要提高包括支气管内超声(endobronchial ultrasound, EBUS)和导航支气管镜，胸腔科医师和胸部外科医师均可操作。这些临床领域的进展在该系列的另一篇文章中已有介绍，这里就不再讨论。

### 术中超声定位肺结节

实施胸腔镜切除时定位肺结节的难易程度取决于病变的大小和部位。传统上，定位中央型、周围型肺结节的金标准是触诊，这需要借助于开胸术。在胸腔镜下无法进行触诊，这是一个固有缺陷。胸腔镜下对比较困难的结节进行操作时，提高成功率的其它选择包括通过如导航支气管镜等方法进行图像引导下针吸定位、基准标记安置或者墨汁染色法。这些方法的潜在缺陷包括额外的术前准备时间、气胸的风险、针头移位或分离术侧肺时发送移位以及靶向肺区域的非特异性墨水染色。

在电视辅助胸腔镜手术(video-assisted thoracoscopic surgery, VATS)中，术中超声已成为术中检测肺结节的一个切实可行的选择。利用一个可弯曲的腹腔镜10 mm超声探头(B-K Medical, Herlev, Denmark)和用于肺检查的导电膏，传统VATS技术检测不到的病变均可被检测到并定位。一旦识别，借助于标准楔形切除技术可将病变切除。介绍术中超声应用的文章在逐渐增多^[1-3]^。一项研究共纳入54例连续的接受经胸廓超声辅助VATS的患者，共定位和切除65个肺结节，报告显示术前由计算机断层扫描诊断的65个肺结节中的16个结节单独通过VATS无法检测到或不明显。术中超声可定位并识别该16个结节中的15个结节^[3]^。该技术的潜在缺陷非常小，包括操作者学习曲线，患者的风险更小，肺结节的增强定位或可使很多患者免受不必要的开胸术。

## 肺癌外科分期的进展

在NSCLC的治疗中，精确的纵隔淋巴结外科分期至关重要。除了避免不必要的手术，精确的外科分期有助于筛选可从新辅助治疗中获益的患者，并利于精确比较不同的临床研究。新辅助治疗后的重新分期对于确定手术切除获益的患者群非常重要。传统上，纵隔镜是NSCLC外科分期的金标准。用于分期的非外科技术仍然存在，包括EBUS联合经支气管针吸活检，并在继续接受检验。用于NSCLC外科分期的不断发展的技术，包括电视辅助纵隔淋巴结清扫术(video-assisted mediastinal lymphadenectomy, VAMLA)和经子宫颈纵隔淋巴结清扫术(transcer vical extended mediastinal lymphadenoctomy, TEMLA)，进一步提高了NSCLC患者分期的有效性和精确度。

### 经子宫颈纵隔淋巴结清扫术

目前有关电视辅助纵隔镜检查术和TEMLA在外科分期中的最新进展为胸部外科大夫提供了更为有效的外科工具，但有一定代价(TEMLA略微增加喉返神经损伤的风险)。TEMLA可完全清除所有淋巴结站点的纵隔淋巴结，为分期提供了最完全、最精确的方法。由于操作复杂性和潜在并发症的增加，不同机构间其适应症也不同^[4]^。在一项最大样本量的研究中，患者(*N*=698)接受TEMLA进行NSCLC分期，其检测N2/3疾病的敏感性为96.2%。在随后继续接受开胸术的445例患者中，仅在7例(1.6%)患者中发现遗漏的N2疾病。阴性预测值为98.7%。据报道该操作总的发病率为6.6%，喉返神经麻痹仅发生于16例(2.3%)患者中。无致死性术中并发症报道。该研究中的5例死亡病例无一归因于TEMLA操作本身^[5]^。

对于伴有高风险纵隔淋巴结受累的患者，我们推荐使用TEMLA。以下患者亚组使用TEMLA具有优势：肿瘤较大的患者、影像学显示肺门淋巴结受累的患者、双侧肺均有原发肿瘤的患者以及计划性切除前已接受新辅助治疗的患者。在接受新辅助化疗或放化疗患者的重新分期方面，报道显示TEMLA优于纵隔镜、EBUS、以及/或PET/ CT的标准重新分期^[6]^。展望未来，用于外科分期的方法和技术将会被进一步完善，并以互补方式被继续应用。例如，EBUS可对可疑性N2疾病进行诊断并分期，使患者无须接受纵隔切除，这对接受新辅助治疗后的将来生活尤为重要。

## 非小细胞肺癌外科治疗进展

### 电视辅助胸腔镜手术的扩大指征

胸腔镜用于早期NSCLC的手术切除已被广泛接受，随着经验的积累，胸腔镜的指征正扩大至更晚期的NSCLC患者。初期围手术期结果显示既安全又可行，胸腔镜正逐渐被应用于复杂的手术切除^[7]^。一项单机构研究报告了局限性晚期NSCLC患者同时需要接受肺切除的VATS方法和结果^[8]^。研究对胸腔镜下胸壁切除术做了介绍，有经验的VATS外科医师可安全操作。该研究的长期结果尚未得知，而且微创技术用于广泛性胸壁切除的作用亦未明。

目前逐步完善的技术和设备也可发挥等效的作用，有时良好的外科切除和暴露可模拟开放手术。通过一个10 mm的切口，低剖面5 mm的仪器可允许多种操作设备。应用于其它外科分支专业的可分离骨结构的工具已被用于NSCLC的胸腔镜下胸壁切除。

### 机器人辅助肺切除术

在过去的几十年里，NSCLC外科治疗的另一最新进展是通过da Vinci外科系统(Intuitive Surgical, Sunnyvale, Califounia)整入机器人辅助外科技术。尽管在普胸外科手术中并未被广泛接受或整入，但确定在其它外科专业机器人辅助所带来的优势能否转入NSCLC的机器人辅助肺切除的兴趣正在逐渐高涨。

通过克服传统胸腔镜切除的一些缺陷，如设备的二维图像、不稳定摄像平台以及通过非肋骨扩展的切口时所使用设备的可操作性较差，机器人辅助肺切除有助于提高现有微创切除的预后。尽管报告仅限于选定的几个机构，但早期结果显示机器人辅助既可行又安全^[9]^。一项纳入接受机器人肺叶切除NSCLC患者的多中心回顾性分析显示，早期NSCLC可进行机器人肺叶切除，发病率和死亡率均较低。基于分期的长期生存可接受，且与接受传统开胸术或VATS切除患者的结果一致^[10]^。

### 单切口胸腔镜手术

尽管大量文献表明VATS可降低肺切除围手术期的发病率，但微创技术更大的优势在于改善患者对肺切除的耐受程度。通过限制单一切口实施切除所需的切口数量，可能降低围手术期发病率。有关单一切口楔形切除、解剖性肺叶切除甚至单一切口VATS肺切除的报告已有介绍^[11-13]^。最近报道了1例接受双侧VATS的病例，经由单一子宫颈切口接近所有区域而不侵犯胸壁^[14]^。尽管从技术层面讲是可行的，但该方法的可能获益尚未得到验证。

技术层面的进步使通过单一切口实施肺切除成为可能，这些进步包括低剖面5 mm胸腔镜设备(Sontek, Columbus, Ohio)、直径为10 mm和5 mm的可调节尖端摄像技术(Olympus)以及可调节尖端内镜吻合技术。

### 亚肺叶切除的新技术

尽管对于早期NSCLC患者的试图根治性切除来说，解剖性肺叶切除术是被广泛接受的金标准，但多数患者心肺功能储备不足以安全经受解剖性肺叶切除术。回顾性分析显示，与接受肺叶切除术患者相比，接受楔形手术患者的局部复发率升高且无病生存降低。一项评估肺叶切除对比亚肺叶切除治疗小的周围型肿瘤(< 2 cm)的全国性随机双盲多中心试验显示，与亚肺叶切除相比，肺叶切除降低了患者的获益。

与简单的楔形切除相比，解剖性肺段切除术使小的NSCLC可以接受肺切除，且预后更好。目前识别肺段支气管中的血管和气道结构的技术的改善使胸腔镜下安全实施亚肺叶解剖性切除成为可能。帮助外科大夫识别肺段解剖的新技术已有介绍，这些新技术包括通过纤维支气管镜进行选择性术中通气。可区别需切除的膨胀与需保留的不膨胀部分。一项研究共纳入52例 < 2 cm的外周型T1肿瘤患者，所有患者均可通过肺段切除术安全接受手术，无死亡病例，总的并发症发生率为13.5%^[15]^。

### 亚肺叶切除联合短程放射治疗

对于因为心肺合并症不能耐受解剖性肺叶切除的患者来说，可延长无病生存的另一选择是亚肺叶切除联合术中短程放射治疗。一项报告比较了亚肺叶切除与亚肺叶切除联合短程放射治疗，结果显示亚肺叶切除联合短程放射治疗能明显降低局部复发。放射性粒子以预先包装形式存在，在VATS楔形切除时可通过外科方便地植入，并且相对安全。一项新近的纳入222例患者的多中心Ⅲ期随机试验显示，与单独亚肺叶切除相比，高风险患者可安全接受亚肺叶切除联合短程放射治疗，在30天和90天时并未增加发病率^[16]^。

### 清醒状态下实施胸外科手术

2004年第一次提到可在清醒状态下通过胸腔镜手术切除孤立性肺结节，目前在多种肺病理类型中有涉及^[17-20]^。可以预料，不需要全身麻醉，双腔气管插管、正压通气会使手术时间缩短，有利于快速恢复。在一项研究中，60例患者被随机分成两个治疗小组，30例接受硬脑膜外麻醉患者的麻醉满意分数较高，住院时间缩短至1天。无死亡病例^[17]^。另一项单独的研究中，在未进行全身麻醉的前提下，46例外周型肺结节患者接受3 mm针式胸腔镜辅助楔形切除术^[18]^。仅两例患者需要重新进行全身麻醉。指征扩展至包括转移灶切除术，在清醒、不进行全身麻醉的状态下实施胸腔镜下肺叶切除术已有报道^[19, 20]^。对于在特定状态下先前认为不宜手术的患者来说，清醒状态下的胸外科手术使其可接受切除术，且发病率较低。

## 晚期疾病的新兴外科技术

### 肺弥漫

不可治愈的Ⅳ期NSCLC患者的长期生存十分不理想。从传统意义上来说，手术可通过胸膜固定术缓解恶性胸腔积液，通过各种方法治疗继发于肿瘤效应的气道阻塞，这些方法包括激光、内镜下冷冻疗法以及具有自膨式支架的气道支架。

全身性化疗的进展也有报道，但治疗的全身疗效对患者来说仍然很恐怖。通过对肺弥漫实施区域性化疗，肿瘤负担主要位于单侧胸廓的患者在治疗时可选择早期剂量递增方案。对肺弥漫通过胸腔镜识别上、下肺静脉，同时将介入治疗血管闭塞球囊置于主肺动脉，通过首次堵塞血管气囊引流目标肺。然后肺静脉萎缩，在预定的时间段在肺内进行区域性化疗，并扩散至周围组织。这并不是持续性扩散。30 min后，肺重新灌注，从而冲洗受治肺。剂量递增研究的初期结果显示非常安全，受治肺的全身效应降低^[21]^。

## 结语

手术方法和技术的最新进展已延伸至NSCLC外科诊断、分期和治疗的选择，同时改善了与这些治疗相关的围手术期发病。
